# Emergence of the Middle East Respiratory Syndrome Coronavirus

**DOI:** 10.1371/journal.ppat.1003595

**Published:** 2013-09-05

**Authors:** Christopher M. Coleman, Matthew B. Frieman

**Affiliations:** Department of Microbiology and Immunology, University of Maryland School of Medicine, Baltimore, Maryland, United States of America; Columbia University, United States of America

## Introduction

It began routinely enough. A patient with severe respiratory disease at the Dr. Soliman Fakeeh Hospital in Jeddah, Saudi Arabia was getting worse and no one knew why. A sample of sputum was sent to Dr. Ali Mohamed Zaki to identify the culprit, as he had identified these diseases many times before. However, this time would be different. The sample showed no positive hits on any of the virus assays he normally used. He contacted Dr. Ron Fouchier, at Erasmus Medical College in Rotterdam, Netherlands, to see if he could be of help. Dr. Zaki's initial idea was that the virus was a paramyxovirus, and Dr Fouchier had recently published a Pan-paramyxovirus polymerase chain reaction (PCR) assay [1]. In Dr. Fouchier's lab, the virus was identified as a novel coronavirus, one that had never been seen before.

This novel coronavirus, now called Middle East respiratory syndrome coronavirus (MERS-CoV), has been identified in several countries across the Middle East and Europe, with primary infections found in Saudi Arabia, Qatar, Jordan, and The United Arab Emirates (UAE) (http://www.who.int/csr/disease/coronavirus_infections/en/). Infections in the United Kingdom, Tunisia, France, Italy, and Germany have been imported by travel from the Middle East. The Italian cluster is believed to be from a patient traveling to Jordan and back, and the French cluster originated from a patient traveling to the UAE. The largest cluster of cases, 23 in total, is in Saudi Arabia. As of July 25, 2013, there are 90 confirmed infections, of which 45 have resulted in death, resulting in a 50% case fatality rate. MERS-CoV has been sequenced from nine infected individuals, and its genome sequence places it in the same sub-family (Group 2) as SARS coronavirus (SARS-CoV), but in a new lineage (called Group 2c) (sequences reported in [2]–[4] and at http://www.hpa.org.uk/webc/HPAwebFile/HPAweb_C/1317138176202; http://www.ncbi.nlm.nih.gov/nuccore/KC776174)(http://www.virology-bonn.de/index.php?id=46).

## What Is the Name of This Novel Coronavirus?

The initial name of this novel coronavirus was hCoV-EMC, which stood for human coronavirus–Erasmus Medical College, where the first isolate was sequenced [3]. An additional isolate, provisionally named human coronavirus England 1, was isolated from a patient in London, UK, who had been flown from Qatar to London for treatment [4]. A report from the Coronavirus Study Group of the International Committee on Taxonomy of Viruses (ICTV) has proposed naming this virus Middle East respiratory syndrome coronavirus (MERS-CoV) [5]. MERS-CoV is provisional until ratified by the ICTV.

## Where Did MERS-CoV Come From? Is There a Natural Reservoir?

The closest phylogenetic neighbors to MERS-CoV are putative bat coronaviruses in China (BtCoV-HKU4 and BtCoV-HKU5) [3], the Netherlands (BtCoV/VM314/2008} [2], and a recently discovered isolate from South Africa [6]. All four of these bat coronaviruses have been sequenced only from bat samples and have never been isolated as live viruses from either bats or the environment. The natural reservoir of MERS-CoV has not been identified, although its similarity to these other four viruses suggests that it is of bat origin. Importantly, SARS-CoV emerged from bats as well [7]. Anecdotal evidence suggests that MERS-CoV may have been transmitted to humans via livestock (camels or goats); however, there is no scientific data yet to support this theory.

## Does MERS-CoV Share Any Features with SARS-CoV beyond the Zoonotic Origin?

Given the similarities in emergence and apparent zoonotic origins between MERS-CoV and SARS-CoV, initial experiments on MERS-CoV focused on direct comparison with the known molecular biology of SARS-CoV. Infection experiments in cell culture showed that MERS-CoV does not use the SARS-CoV receptor, angiotensin converting enzyme 2 (ACE2), for entry, and that MERS-CoV has a much broader host range than the epidemic isolate of SARS-CoV [8]–[14]. The genome structure of MERS-CoV is similar to other coronaviruses, with the 5′ two-thirds of the genome encoding the non-structural proteins (NSPs) required for viral genome replication, the remaining 3′ third of the genome encoding the structural genes that make up the virion (spike, envelope, membrane, and nucleocapsid proteins), and four accessory genes interspersed within the structural gene region [2]. One additional similarity between MERS-CoV and SARS-CoV is their abilities to inhibit a robust type I interferon (IFN) response in infected cells. However, MERS-CoV has been shown to be much more sensitive to exogenous type I IFN treatment compared to SARS-CoV, which may be important for pathogenesis [8], [11], [14], [15]. Several SARS-CoV-encoded proteins have demonstrated innate immune signaling antagonism [16], and MERS-CoV encodes several IFN antagonists as well (Matthews et al, submitted, Muller et al, submitted).

## What Is the Receptor for MERS-CoV and What Cells Does It Infect?

MERS-CoV has been shown to infect a range of human, primate, porcine, and bat cell lines [11]. *Ex-vivo* infections of human lungs and human airway epithelial cell cultures identified type II alveolar cells and non-ciliated lung epithelial cells (Clara cells) as the targets of infection, rather than the ACE2-expressing ciliated epithelial cells that SARS-CoV targets [9], [15]. Interestingly, in at least one case, endothelial cells were infected as well [15], showing a distinct difference between the biology of SARS-CoV and MERS-CoV, as SARS-CoV specifically infects ciliated epithelial cells in the lung [17], [18]. The receptor for MERS-CoV was recently identified as dipeptidyl peptidase 4 (DDP4) by mass spectrometry analysis of Huh7 cell protein bound to the MERS-CoV Spike protein *in vitro* [10]. Transfection and localization experiments demonstrated that DPP4 is indeed the receptor for MERS-CoV and is necessary for infection of a non-permissive cell line [10]. DPP4 has many diverse functions in glucose homeostasis, T-cell activation, neurotransmitter function, and modulation of cardiac signaling [19]. ACE2 does not require enzymatic function in order to act as a receptor for SARS-CoV entry, but the enzymatic function of ACE2 has been linked to severity of the disease following SARS-CoV infection [20]. Similarly, inhibition of the enzymatic function of DPP4 did not affect virus entry *in vitro*; however, the role of DDP4 enzymatic activity has not been investigated *in vivo* [10].

## What Is the Host Response to MERS-CoV?

Transcriptional analysis of MERS-CoV infected cells has identified several pathways specifically modulated during infection [9]. MERS-CoV is shown to modulate the innate immune response, antigen presentation, mitogen-activated protein kinase (MAPK), and apoptosis pathways. Inhibition of the MAPK pathway showed reduction in viral replication in culture, pointing to potential therapeutics. Importantly, several studies show that MERS-CoV, similar to SARS-CoV, does not induce an early type I IFN response, suggesting that MERS-CoV may encode proteins that inhibit sensing of the viral RNA during infection [8], [11], [14], [15]. The modulation of these pathways may explain the increased lethality of MERS-CoV.

## Is There a Small Animal Model of MERS-CoV for the Study of Pathogenesis?

There is currently no small animal model for MERS-CoV. Rhesus macaques infected with MERS-CoV display pneumonia, reduced appetite, significant lung pathology, and inflammatory infiltrates [21]. However, MERS-CoV does not replicate in BALB/c, C57B/6, 129SvEv, or STAT1 knockout mice on the 129SvEv background (Coleman et al, submitted). Interestingly, mouse DPP4 is highly similar to the human DPP4, varying at only 62 positions out of 767 amino acids residues total (92% similarity). However, the differences tend to be on surface-exposed residues which, therefore, could affect binding of viral spike protein to mouse DPP4. Future structural and functional interaction experiments are needed to identify if the mouse DPP4 interacts differently with MERS-CoV spike, as compared to human DPP4, and if the known mutations allowing for this binding could be used for the development of a mouse model of MERS-CoV.

## Are There Approved Treatments or Vaccinations for MERS?

There is no current treatment or vaccination available for MERS-CoV, but, with the continuation of the outbreak, identification of therapeutics is a top priority. Several manuscripts have demonstrated that a variety of therapeutics inhibit MERS-CoV replication in cell culture [9], [15]. None have been tested *in vivo*, in part due to the lack of a small animal model, as described above. One promising avenue is to use the knowledge of SARS-CoV and compare it to MERS-CoV. IFNα was shown in multiple models to protect against SARS-CoV-induced disease. MERS-CoV is also sensitive to IFNβ treatment *in vitro* [15]. Ribavirin, a known inhibitor of RNA viruses, has also been demonstrated to inhibit MERS-CoV replication, and together they can inhibit MERS-CoV at nanomolar levels [22]. Other inhibitors were shown to affect specific pathways, specifically the MAPK pathway. The MAPK inhibitor SB203580 was shown to inhibit MERS-CoV replication in VerE6 cells [9]. Additional therapeutics and vaccinations are in development, with a focus on FDA compounds already in use.

## Conclusion and Questions

Many unanswered questions remain on this newly identified virus:

1. What is the environmental reservoir of MERS-CoV? Is it transmitted from bats to camels, goats, or cats? Is the virus linked to date palm harvesting? How did it spread to people from the environment?
2. Are there associated comorbidities that predispose someone to contracting MERS? With the age of infected patients skewed toward older males, is there a genetic link to infection? Are the patients generally immunosuppressed?
3. What is the seroprevalence of MERS-CoV in the general population? Has MERS-CoV been circulating for many years between animals and people and only now mutated enough to be able to cause disease in people? Or is this a new spillover event that has not been seen by humans until now?
4. Why doesn't MERS-CoV grow in mouse cells or cause disease in mice? Is it because the viral spike protein doesn't bind mouse DPP4 at all, is it because there are other host factors necessary for entry and replication in mouse cells, or is it due to location and amounts of receptor expression?
5. How do the MERS-CoV proteins contribute to disease? Are there any specific functions of the proteins that allow for enhanced pathogenesis?
6. Since this virus is similar to bat coronaviruses identified in China, Africa, and Europe, why haven't other bat coronaviruses spilled over into people, causing serious disease (with the exception of SARS-CoV [7] and, potentially, hCoV-229E [23])? What is it about MERS-CoV and the conditions in the Middle East that have contributed to viral infection and the high mortality rate?

With the spread of MERS-CoV through the Middle East, one thing is certain at this point: The emergence of the novel SARS coronavirus in 2003 from a zoonotic source in China and its spread around the world is not an isolated incident of coronavirus spread. Continued spillover events will occur from animals to humans in the future. The sooner we understand these current microbial threats, the more people we can save from infection and possible death. If we can identify these microbes in our environment before they infect us, we can better protect ourselves against future infections.
